# Development of the Paranormal and Supernatural Beliefs Scale using classical and modern test theory

**DOI:** 10.1186/s40359-021-00600-y

**Published:** 2021-06-23

**Authors:** Charlotte E. Dean, Shazia Akhtar, Tim M. Gale, Karen Irvine, Richard Wiseman, Keith R. Laws

**Affiliations:** Department of Psychology, Sport and Geography, School of Life and Medical Sciences, University of Hertfordshire, Hertfordshire, UK

**Keywords:** Paranormal beliefs, Anomalous beliefs, Supernatural, Scale, Scale development, Factor analysis, Rasch analysis, Rating scale model, Classical test theory, Modern test theory

## Abstract

**Background:**

This study describes the construction and validation of a new scale for measuring belief in paranormal phenomena. The work aims to address psychometric and conceptual shortcomings associated with existing measures of paranormal belief. The study also compares the use of classic test theory and modern test theory as methods for scale development.

**Method:**

We combined novel items and amended items taken from existing scales, to produce an initial corpus of 29 items. Two hundred and thirty-one adult participants rated their level of agreement with each item using a seven-point Likert scale.

**Results:**

Classical test theory methods (including exploratory factor analysis and principal components analysis) reduced the scale to 14 items and one overarching factor: *Supernatural Beliefs*. The factor demonstrated high internal reliability, with an excellent test–retest reliability for the total scale. Modern test theory methods (Rasch analysis using a rating scale model) reduced the scale to 13 items with a four-point response format. The Rasch scale was found to be most effective at differentiating between individuals with moderate-high levels of paranormal beliefs, and differential item functioning analysis indicated that the Rasch scale represents a valid measure of belief in paranormal phenomena.

**Conclusions:**

The scale developed using modern test theory is identified as the final scale as this model allowed for in-depth analyses and refinement of the scale that was not possible using classical test theory. Results support the psychometric reliability of this new scale for assessing belief in paranormal phenomena, particularly when differentiating between individuals with higher levels of belief.

**Supplementary Information:**

The online version contains supplementary material available at 10.1186/s40359-021-00600-y.

## Background

Research suggests that belief in the paranormal correlates with a range of psychological constructs, including anxiety, locus of control, suggestion, imagery, fantasy proneness, critical thinking, religiosity and creativity [1–7]. Belief in the paranormal has also been shown to correlate with cognitive factors such as cognitive ability, thinking style and executive function [8–11]. Similarly, associations have been seen between academic discipline and paranormal beliefs, particularly when comparing hard science and medical students to those from the arts and humanities [8, 12, 13], although some ambiguity surrounds these findings [2, 14]. Finally, some evidence indicates that demographic characteristics such as age and gender also influence belief in the paranormal, although the extent of these effects have been questioned [15–18]. While much of this work indicates a negative influence of paranormal beliefs on cognition and psychological well-being, many studies have also demonstrated positive and adaptive functions of such beliefs. These adaptive functions include goal setting, emotional clarity, clarity about the self and the wider world, coping with trauma and stress, and the reduction of fear surrounding ambiguous stimuli [19–26]. Similarly, paranormal experiences have been shown to have adaptive outcomes, particularly in the wake of a bereavement [27–30]. These positive experiences may in turn lead to belief in the paranormal. Indeed, several studies have reported positive correlations between paranormal experience and belief [31–33]. This may also relate to the relationship between emotion-based reasoning and an individual’s proneness to paranormal attributions [34, 35]. Regardless of the cause of these beliefs, the breadth of work in this area suggests that belief in the paranormal should not be automatically viewed as a negative or problematic trait. However, some researchers argue that there is a specific type of believer whose beliefs are more likely to be associated with negative biases and dysfunctions. Previous work has suggested that paranormal believers can be divided into two subgroups: informed believers (who have a deeper understanding of paranormal phenomena and their putative causes), and quasi-believers (whose beliefs represent a superficial understanding of paranormal phenomena) [36, 37]. It has been proposed that negative associations seen between paranormal beliefs and cognition are a function of a tendency to hold quasi-beliefs, and that informed believers represent a small subgroup of believers whose beliefs are independent of any cognitive deficits [36, 37]. However, it is still unclear whether paranormal believers can be reliably divided into such subgroups [38].

Despite this large amount of work, researchers have yet to agree on a definition of the term “paranormal”. While a review of existing definitions is beyond the scope of this paper, the present work adopts the widely held view that phenomena can be considered paranormal when they violate the basic limiting principles of current scientific understanding [39], and so includes phenomena such as telepathy, life after death, astrology, and hauntings.

However, widespread agreement exists that research in this area is hampered both by studies employing a diverse range of measures of paranormal belief [4, 40–44], and by the lack of psychometric validity for some scales [45–48]. Much of the discussion has focussed on the three most frequently used scales—namely, the Paranormal Belief Scale [49], the Australian Sheep-Goat Scale [50] and the Survey of Scientifically Unaccepted Beliefs [51].

### Paranormal Belief Scale

The Paranormal Belief scale in both original [49] and revised format (RPBS) [52] is the most widely used measure of paranormal belief. The revised format contains 26 items, adopts a broad definition of paranormal phenomena, and contains seven subscales (Traditional Religious Belief, Psi, Witchcraft, Superstition, Spiritualism, Extraordinary Life Forms, and Precognition). Several issues have been raised regarding both the item content and the factor structure of the RPBS [47, 48, 53–61]. Much of this criticism has centred on the Extraordinary Life Forms (ELF) and Traditional Religious Belief (TRB) subscales.

The ELF subscale consists of several cryptozoological items, including those relating to the alleged existence of the Loch Ness monster and the abominable snowman of Tibet. Some have argued that endorsing the existence of such alleged extraordinary life forms is not strongly associated with belief in more ‘mainstream’ paranormal phenomena, such as telepathy and premonitions [47]. These cryptozoological items have also been shown to be problematic in samples with greater cultural diversity, leading some researchers to replace items with more culturally relevant equivalents [62–65]. The ELF subscale also has the lowest internal reliability of the seven RPBS subscales and has frequently failed to reach recommended Cronbach’s alpha thresholds [53, 66–69].

The TRB subscale has raised concerns due to contradictory evidence concerning the relationship between paranormal and religious beliefs. While several studies have noted positive correlations between religiosity and belief in the paranormal [9, 70, 71], others have found those displaying especially strong forms of religious belief to be less likely to endorse the existence of paranormal phenomena [72, 73]. Some suggest that the relationship may be best conceptualised as curvilinear, with paranormal belief increasing alongside religious beliefs, but then decreasing when religious beliefs become particularly strong [74, 75].

A further criticism of the RPBS has focused on the fact that only one item in the scale is negatively worded. This could clearly increase the risk of RPBS scores being affected by respondents endorsing this item without fully considering its content [76].

### Australian Sheep-Goat Scale

The Australian Sheep-Goat Scale (ASGS) [50] consists of 18 items and contains three subscales (Belief in Extrasensory Perception, Psychokinesis, and Life After Death). The original response format for the ASGS involved a visual analogue scale, with respondents indicating their level of agreement with each item by marking a horizontal line. Scoring involved using a ruler to yield a value from 1 to 44, and these scores were then recoded to give a final value of 0, 1 or 2 for each item. Subsequent versions employed a force-choice format, with participants selecting one of three response options (‘True’, ‘Uncertain’, and ‘False’) that are then recoded as 2, 1 or 0. The visual analogue scale and forced choice options produce similar overall scores [77]. The ASGS has also been adapted for use with a six-point Likert scale, with some authors arguing that this format is less confusing for respondents and easier to interpret than the original visual analogue [78].

Although the ASGS tends to yield moderate-to-large intercorrelations between the three subscales, the *Life After Death* subscale exhibits the lowest internal reliability, leading some to suggest that it may undermine overall scale integrity [45]. Also, although the visual analogue ASGS presented both negatively and positively worded items, the more frequently employed force-choice and Likert formats lack any negatively phrased items. As such, they raise concerns about response bias.

Despite the issues raised with the ASGS and its differences to the RPBS, several studies have noted positive correlations of 0.70 and above between the two scales [79, 80].

### Survey of scientifically unaccepted beliefs

The Survey of Scientifically Unaccepted Beliefs (SSUB, also referred to as the “Survey of Popular Beliefs”) [51] is a more recent alternative for measuring belief in the paranormal. The SSUB is made up of 20 items and contains two subscales: New Age Beliefs and Traditional Religious Beliefs. The scale has high levels of internal reliability [51, 81, 82], has a balance of positive and negative items, and has not seen the same level of scrutiny and critique as the ASGS or RPBS. Although many of the phenomena featured in the scale could be considered paranormal (e.g., the existence of genuine haunted houses, psychics and fortune tellers), the inventory also contains items relating to several scientifically unaccepted beliefs that are not commonly associated with the paranormal (e.g., the lack of a rational explanation of crop circles and pixies, which are based upon mystery and elusiveness rather than a strict violation of scientific principles).

### Differential item functioning

In addition to the criticisms outlined above, some researchers have questioned whether variations in responses on the existing scales may be partly a function of semantic biases introduced by age or gender, rather than fluctuations in level of belief [56]. This issue is commonly referred to as differential item functioning (DIF). Rasch scaling (a modern test theory model) has been applied to the ASGS [83] as a way of detecting these biases and assessing their effect. Findings indicated weak age and gender biases for some ASGS items, but the effect of these biases was minimal and suggests that the scale is not significantly affected by DIF. The same scaling has also been applied to the RPBS [56], with significant DIF for gender seen on 18 items, and age on 15 items. Consequently, using top-down purification (combining factor analysis and Rasch scaling), a two-factor model was suggested to reduce the impact of DIF, which has subsequently been employed in several studies [22, 33, 34, 84–86]. Despite the extensive use of the purified scale, several items of the RPBS failed to load on either of the two new factors, with the authors highlighting that addition of new items to the RPBS may produce additional belief clusters to those identified through their analyses [56]. DIF analysis was also used in the construction of the SSUB to remove three items from the original item pool that were identified for age and gender biases [51]. As such, these items do not feature on the final version of the SSUB.

### Classical test theory and modern test theory

Latent traits such as paranormal beliefs are, by definition, unobservable. Therefore, research relies on the use of self-report scales, like those mentioned above, which assume that individuals’ responses to items are influenced by the latent trait of interest [87]. Classical test theory (CTT) and modern test theory (MTT; also referred to as item response theory) are the two primary methods used in psychological scale development. Both CTT and MTT models strive to measure and improve the reliability, validity, and internal consistency of the scale under assessment [88, 89] but do so in different ways. One of the key differences between these approaches is that CTT assumes that measurement precision is equal for all individuals, while MTT takes the view that measurement precision depends on individuals’ levels of the latent trait [90].

CTT models, focused at the test-score level, assume a linear model that links the observable test score (X) to the sum of two unobservable variables: true score (T) and error score (E) [91]. This assumption can be more clearly illustrated with the following formula: X = T + E. In this formula, the observed score (X) represents the observed total score calculated from the scale in use, and the error score represents a random, non-systematic error assumed to be independent of the true score (e.g., poorly functioning test items, or external confounding variables). The true score is often conceptualised as the mean of all scores obtained if an individual responded to the given scale an infinite number of times [92]. Therefore, the observed score of X can be considered to be a combination of both relevant information relating to the latent variable of interest and the error associated with each item [93]*.* A factor-analytic strategy (often relying on the use of exploratory factor analysis for item selection) is among the most popular CTT method for scale development, and has the primary aim of developing an internally consistent scale with a manageable number of differentiable dimensions [94].

CTT models offer certain advantages. For example, many CTT models are based on relatively weak assumptions, and are therefore easily met with real test data [91]. These models are also simple to use and allow for examination at the test-score level of the precision with which the latent trait of interest is measured by a given scale [95]*.* However, CTT’s standing popularity, despite the emergence of more modern approaches to scale development, could be attributed to the fact that many researchers are familiar with its basic concepts and are likely to have encountered CTT (or to have used scales that were developed through CTT methods) [93]. Therefore, it is important to also consider the limitations of CTT. The central limitation of CTT models is that person and item parameters are sample-dependent, which limits the utility of these statistics in scale development [89, 91]. CTT models also do not allow for rigorous assessment of item characteristics that can be computed under different models, and so scales developed using CTT methods may suffer from differential item functioning (as mentioned above) [93].

In contrast to CTT models, MTT models are nonlinear and focus at the item level, seeking to relate respondents’ performance on individual test items to their estimated level of the latent trait of interest [96]*.* These models are assumed to be invariant across populations, meaning the item and test parameters can be interpreted independent of specific samples. The type of MTT model used in scale development may differ depending on the type of data collected (dichotomous data such as yes/no responses, or polytomous data collected using Likert response methods), and on the number of dimensions they specify. In general, MTT models can be said to have three main goals: (1) to produce items that provide the most information about respondents’ levels of the latent trait of interest, (2) to present respondents with items tailored to their latent trait levels, and (3) to reduce the number of items needed to determine respondents’ level of the latent trait without loss of reliability [96]*.* The advantages of MTT models over CTT models are most notable at the item level. Item characteristics, differential functioning and fit to the model can be assessed, as well as individuals’ response styles and the functionality of response scales [97]. However, a limitation of MTT models is their use of sophisticated and in-depth statistical analyses which remain unfamiliar to many researchers and testing professionals [96]. The assumptions of MTT models are also more restrictive compared to those of CTT models (i.e., more difficult to meet with real test data), and sample size requirements are much larger for both items and respondents [97]. For unidimensional MTT models (such as the Rasch model), minimum sample sizes of approximately 200 respondents are required [98]. However, multidimensional MTT models require large sample sizes ≥ 1000 respondents to identify precise item parameters and decrease error estimation [99]*.*

CTT and MTT models both have their individual strengths relating to scale development and assessment. Therefore, complete and successful psychometric assessment may benefit from the use of both models, which would provide information about individual item functioning as well as how items function as a unit [97].

### Present study

Paranormal belief scales suffer from various shortcomings, including sub-scales that are often heavily culture specific or do not reflect mainstream beliefs commonly associated with the paranormal, a lack of negatively phrased items and the potential for differential item functioning. The present study sought to address these issues by creating a scale that included phenomena that are widely considered to be associated with the paranormal, had less culture-bound items, combined both positively and negatively phrased items, and did not contain evidence of differential item functioning. The first aim of this study was to construct a scale for measuring paranormal beliefs, examine the latent structure and refine the scale using both CTT and MTT models. The second aim was to assess the test–retest reliability of the new scale(s). Finally, the study aimed to compare the scales developed through CTT and MTT methods to determine the usefulness of each approach, and to determine which scale provides the most precise measure of belief in paranormal phenomena.

## Method and materials

### Participants

We recruited an opportunistic sample of the general public (N = 343) through advertisements placed on social media. These advertisements asked for participants over the age of 18 and fluent in English to complete several short questions about their beliefs in paranormal and superstitious phenomena, as well as a few short questions about themselves. Removal of incomplete responses resulted in the final sample (N = 231: 83 males and 144 females, 4 unreported: Age 18–80, *M* = 36.94, *SD* = 14.60). Most participants were white (51.10% white British, 21.20% other white background, 06.90% White Irish) and held an undergraduate degree or higher (71.00%). Of the participants with a university education, most had a background in psychology (21.60%).

### Materials

An initial collection of 29 statements regarding paranormal and superstitious phenomena was generated using adapted items from: the Revised Paranormal Belief Scale (RPBS) [52], the Australian Sheep-Goat Scale (ASGS) [50], and the Survey of Scientifically Unaccepted beliefs (SSUB) [51], as well as four novel items developed by the authors. These novel items arose from discussion and examination of the RPBS, ASGS and SSUB to identify any phenomena absent from these measures, such as possessions and protection objects. Examples of the phenomena used include luck (lucky charms and bad luck), psi (sixth sense and psychics) and hauntings (Ouija boards and possession). The scale contained both positively (n = 23) and negatively phrased items (n = 6).

### Procedure

The scale was administered as an online survey using Qualtrics Survey Software (Qualtrics, Provo, UT; see https://www.qualtrics.com). Participants were informed that the study was concerned with paranormal and superstitious belief within the general population. Respondents who agreed to take part were asked to provide their age, gender (male, female, other), ethnicity (Arabic, Asian/Asian British, Bangladeshi, Black/Black British, Chinese, Indian, Pakistani, White British, White Irish, other Asian background, other White background, mixed background) level of education (doctoral degree, postgraduate degree, undergraduate degree, post-secondary education, secondary education, vocational) and academic discipline if they had indicated a university education (architecture, arts and humanities, business, education, law, medicine, natural sciences, philosophy, psychology, social sciences, theology, technology, other medical, other). Respondents had the option not to provide the above demographic details. Participants then completed the paranormal scale. Responses were recorded using a 7-point Likert scale (Strongly Disagree, Moderately Disagree, Slightly Disagree, Uncertain, Slightly Agree, Moderately Agree, Strongly Agree). The seven response options were numerically coded from 1 to 7 for positively worded items, and reverse coded for the negatively worded items. Following completion of the scale, we asked participants if they would be willing to complete the scale again one-week from the date of initial completion.

Informed consent was obtained from all participants and all methods were performed in accordance with relevant guidelines and regulations. Ethical approval for the study was granted by the University of Hertfordshire Health, Science, Engineering and Technology Ethics Committee with Delegated Authority (HSET ECDA).

### Data analysis

Analyses will be conducted using two models: a classical test theory (CTT) model and a modern test theory (MTT) model. Therefore, the analysis will use both an exploratory factor analysis (EFA) and a rating scale model (Rasch model). The EFA will allow for the identification of underlying latent constructs underpinning the scale. In other words, EFA will be used to identify emerging sub-categories (or factors) across the initial collection of 29 items. Factors emerging through EFA will be interpreted as distinct categories of paranormal belief. EFA will be conducted using a principal components extraction method, selecting only eigenvalues greater than 1, and a direct oblimin rotation. Items with factor loadings < 0.50 will be removed from the scale and the EFA run again until all items have acceptable factor loadings. EFA will also explore group differences and answering patterns to the scale items and factors to further assess the effectiveness of the remaining scale items.

Rasch analysis will be conducted to allow for a comparison between CTT and MTT methods of scale development. Owing to the polytomous nature of the data, a rating scale model (RSM) [100] will be adopted for the Rasch analysis. Analyses will first evaluate item thresholds and item characteristic curves (ICCs) for the initial collection of 29 items to assess the suitability of the 7-point Likert response format. Item fit to the model will then be assessed by examining both infit (weighted) and outfit (unweighted) mean square statistics (MNSQ). Items identified for overfitting (MNSQ < 0.07/t < -2) or underfitting/misfitting (MNSQ > 1.2/t > 2) will be removed from the scale [101]. The person-item map will then be consulted to assess item difficulty, and to determine whether the remaining items meaningfully measure the ability (level of belief) of all persons. Therefore, we will be using the person-item map to determine whether the final scale is suitable for measuring the range of paranormal belief (from low belief/scepticism to high belief). A CTT method of confirmatory factor analysis (CFA) will be used alongside the Rasch analysis to confirm the unidimensional model fit of the RSM. Finally, remaining items will be tested for DIF in relation to: age, gender, ethnicity, education, or discipline.

A test–retest reliability analysis will be conducted for both the CTT and MTT scales.

## Results: classical test theory

### Factor structure of the scale

An exploratory factor analysis (EFA) was conducted to investigate the latent constructs underpinning the scale. A principal components extraction method was employed and only eigenvalues greater than one were extracted. A direct oblimin rotation was used to account for the non-orthogonality of the items. Bartlett’s Test of Sphericity was significant (*χ*^*2*^ = 4975.77, *p* < 0.001) and the Kaiser–Mayer–Olkin value equalled 0.95 indicating that the data were suitable for further analysis. A four-factor solution was extracted, accounting for 64.32% of the total variance. Cronbach’s Alpha was computed for each factor, with all four showing good internal consistency (α > 0.70). Examination of the pattern matrix revealed seven items with low item loadings (< 0.50), and so a second analysis was undertaken after excluding these items. The second analysis conducted on 22 items indicated a three-factor solution, accounting for 63.94% of the total variance. Inspection of the pattern matrix revealed a further two items with loadings < 0.50, leading to an analysis restricted to 20 of the scale items. The final analysis accounted for 65.67% of the total variance. All emergent factors demonstrated good levels of internal consistency and were conceptually distinct. Of the nine items that were removed during EFA, most were concerned with belief in psychics and those with supernatural abilities (e.g., “psychokinesis, the movement of objects through psychic powers, does exist”, “tarot cards are an accurate way to see a person’s past, present, and future”, “astrology is a way to accurately predict the future”, “mind reading is possible”).

The first factor, eigenvalue 10.07, accounted for 50.34% of the variance and demonstrated excellent internal reliability (α = 0.95). The 14 items contained within Factor 1 concerned phenomena such as spell casting, communicating with the dead, hauntings, possession, the soul, and premonitions. As this factor contained 70% of the total scale items and covered a variety of paranormal phenomena that could be considered supernatural, Factor 1 was subsequently labelled “*Supernatural Beliefs*”. The second factor had an eigenvalue of 1.87 and accounted for 9.34% of the variance. Factor 2 showed excellent internal reliability (α = 0.88). The factor comprised three items concerned with common superstitions centred around bad luck. Factor 2 was subsequently labelled “*Bad Luck*”. The final factor, eigenvalue 1.20, accounted for 5.99% of the variance, with low to moderate internal reliability (α = 0.53). Factor 3 comprised three items regarding telepathy, charms, and predicting the future, and was labelled “*Psi*”.

### Response differences between believers and sceptics

We divided participants into groups of ‘believers’ and ‘sceptics’ according to their mean scores (with those scoring below the overall mean of 67.30 identified as ‘sceptics’ and those above as ‘believers’). The total sample comprised 117 (50.60%) sceptics and 114 (49.40%) believers.

### Principal component analysis

**Fig. 1:**
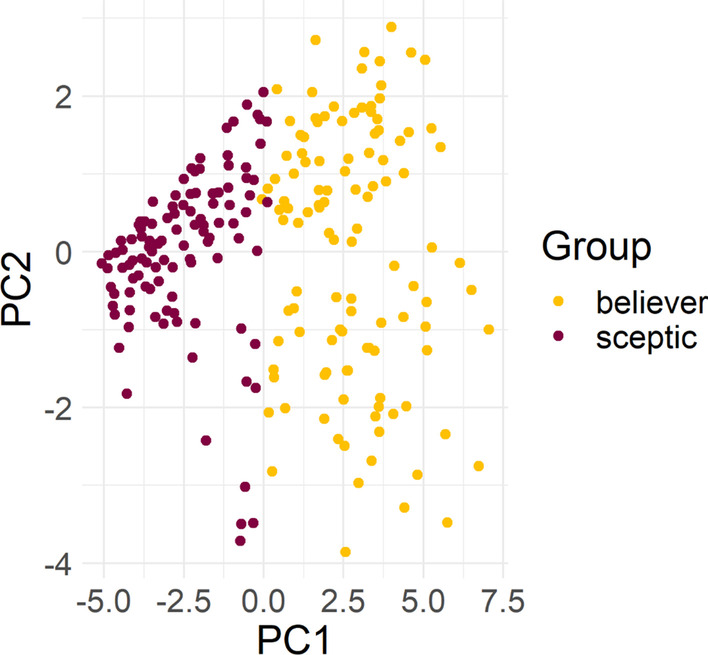
PCA score plot of all responses to the paranormal scale as a function of respondent group. Figure plots participants’ responses to the scale items against the two principal components that represent the largest variability among the two groups, to provide a visual indication of separation (or lack thereof) between the groups

**Fig. 2:**
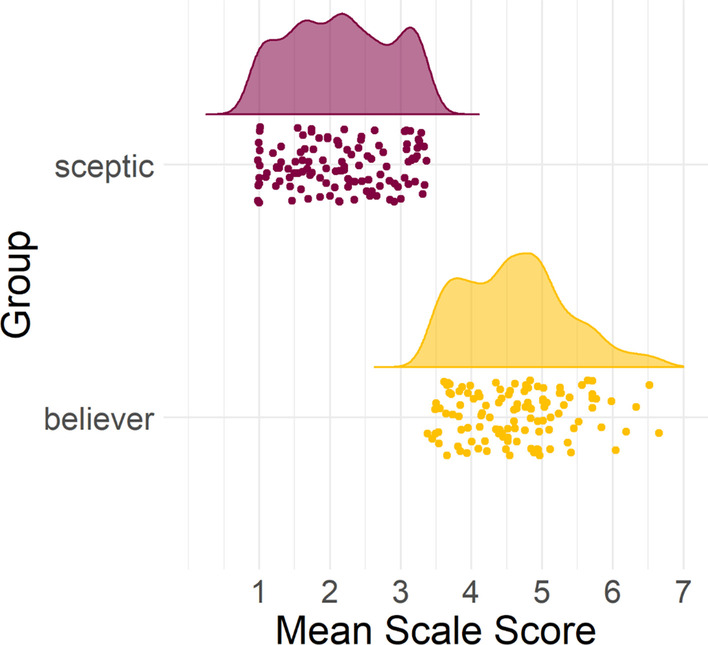
Raincloud plot of mean scale scores given as a function of respondent group. Figure presents mean Likert scores (from 1 to 7) for all items on the scale, with individual mean scores per participant shown for each group, and a histogram showing the distribution of mean scale scores for each group

To provide a visual overview of answering patterns for the two groups, a principal component analysis (PCA) was conducted using the ggfortifiy [102] package in R version 4.0.2 [103]. The PCA score plot (see Fig. 1) shows responses to all 20 items as a function of respondent group, and highlights the distinct clustering of believers and sceptics, with very little overlap between the two groups. To visually represent the responses to each item on the scale for believers and sceptics, a raincloud plot was created, and the results can be seen in Fig. 2.

### Group answering patterns

Responses for believers and sceptics were tested for each item and factor. Table 1 displays the percentage agreement for each item and subsequent factor across both groups. Responses labelled “strongly disagree”, “moderately disagree” and “slightly disagree” were collapsed to give an overall “disagree” score for a given item or factor. The same was done for responses labelled “strongly agree”, “moderately agree” and “slightly agree” to provide an overall “agree” score. Participants’ percentage of “uncertain” responses are also shown here as a function of respondent group. Percentage agreement was also calculated for participants in the upper and lower quartiles to provide a more accurate reflection of item-based differences for the most sceptical participants and those with the strongest paranormal beliefs (see Table 2).

**Table 1:** Percentage agreement with factors and items as a function of respondent group

	Disagree %		Uncertain %		Agree %	
	Believers	Sceptics	Believers	Sceptics	Believers	Sceptics
Factor 1 Total	13	75	19	12	67	13
Item 1	4	60	16	13	80	27
Item 3	8	55	18	22	74	23
Item 5	31	90	25	5	45	5
Item 7	14	71	22	20	64	9
Item 8	11	87	16	5	73	8
Item 9	13	82	9	13	78	5
Item 10	3	64	11	15	87	21
Item 12	8	72	28	17	64	11
Item 13	22	91	11	5	67	4
Item 15*	9	61	24	18	68	21
Item 16	13	68	10	7	77	26
Item 17*	13	80	31	11	56	9
Item 18	20	86	25	6	54	8
Item 20	18	91	26	7	56	3
Factor 2 Total	64	94	12	2	23	4
Item 2	61	92	15	3	25	5
Item 4	60	95	11	3	30	3
Item 6	73	95	11	1	16	4
Factor 3 Total	30	69	20	6	49	25
Item 11*	46	85	14	1	39	15
Item 14*	23	57	24	10	54	32
Item 19*	21	64	24	8	55	28

*Reverse scored items, table presents the percentage of believers and sceptics who indicated agreement, disagreement, or uncertainty for each item and each factor

**Table 2:** Percentage agreement with factors and items for upper and lower quartiles

	Disagree %		Uncertain %		Agree %	
	Upper quartile	Lower quartile	Upper quartile	Lower quartile	Upper quartile	Lower quartile
Factor 1 Total	6	91	11	4	83	4
Item 1	3	82	3	5	93	13
Item 3	2	84	7	8	91	8
Item 5	12	98	19	0	69	2
Item 7	7	85	16	11	78	3
Item 8	2	98	3	2	95	0
Item 9	3	98	0	2	97	0
Item 10	0	92	3	3	97	5
Item 12	2	94	21	6	78	0
Item 13	12	98	12	0	76	2
Item 15*	3	76	19	13	78	11
Item 16	7	82	3	5	90	13
Item 17*	9	94	16	2	76	5
Item 18	10	97	16	2	74	2
Item 20	5	100	17	0	78	0
Factor 2 Total	55	99	15	0	30	1
Item 2	55	98	17	0	28	2
Item 4	45	100	10	0	45	0
Item 6	64	100	17	0	19	0
Factor 3 Total	19	74	23	5	58	22
Item 11*	28	82	17	2	55	16
Item 14*	12	69	24	8	64	23
Item 19*	17	69	28	5	55	26

*Reverse scored items, table presents the percentage of participants in the upper and lower quartiles who indicated agreement, disagreement, or uncertainty for each item and each factor

To test for differences in the two groups, items were then stacked by factor and Chi-Square analysis was conducted. Believers and sceptics differed reliably on all factors, with believers scoring significantly higher than sceptics (i.e., agreeing with more of the statements) for each of the three factors (see Table 3).

**Table 3:** Mean score (standard errors), χ^2^, *p* values, and Cramer’s V for likelihood ratio tests for groups within each factor

Factor	Mean (SE)				
	Believers	Sceptics	χ^2^	*p*	Cramer’s V
1	5.07 (.10)	2.19 (.11)	1330.63	< .001	.45
2	2.82 (.12)	1.41 (.07)	93.24	< .001	.26
3	4.32 (.11)	2.64 (.14)	105.83	< .001	.28

Examination of the group answering patterns revealed that, while most believers agreed overall with Factors 1 and 3, a higher proportion disagreed with Factor 2. Therefore, it can be said that the items in Factor 2 are less effective in separating believers and sceptics, particularly when compared to the percentage scores for Factor 1. Inspection of Table 3 revealed that the scores for believers and sceptics were most similar for Factor 2, with Factors 2 and 3 both displaying small effect sizes. As Factors 2 and 3 both presented limitations (both had small effect sizes, Factor 2 was less effective in separating the two groups, and Factor 3’s internal reliability was below satisfactory thresholds), a final exploratory factor analysis was conducted removing the six items contained within Factors 2 and 3. The analysis used the same extraction and rotation methods as before. Bartlett’s Test of Sphericity was significant (*χ*^*2*^ = 2565.14, *p* < 0.001) and the Kaiser–Mayer–Olkin value equalled 0.95 indicating that the data were suitable for further analysis. A one-factor solution was extracted, accounting for 62.93% of the total variance. Cronbach’s Alpha was computed for this factor, which retained the excellent internal consistency found in the earlier analysis (α = 0.95). Table 4 presents the final 14 items contained within the single factor alongside the component loadings seen in the (non-rotated) component matrix.

**Table 4:** Single-factor scale with corresponding Cronbach’s Alpha (α) score and component loadings

Factor	α	Items (loading scores)
1 Supernatural Beliefs	.95	1 The soul continues to exist after a person has died (.76)
		2 Your mind or soul can leave your body (.77)
		3 It is possible to cast spells on persons using formulas and incantations (.80)
		4 It is possible to be reincarnated (.74)
		5 Some people with psychic abilities can accurately see the future (.86)
		6 It is possible to communicate with the dead (.86)
		7 Buildings can be haunted by spirits or other supernatural entities (.87)
		8 Some psychics have helped find the bodies of murder victims through paranormal means (.85)
		9 A person’s star sign can have a direct influence on their personality (.76)
		10* Reports of an apparent sixth sense are generally based on fantasies (.72)
		11 Having a dream that comes true is not just a coincidence (.71)
		12* Communicating with spirits or other supernatural entities through a Ouija board is not possible (.75)
		13 It is possible to become possessed by an evil supernatural entity (.81)
		14 It is possible to protect one’s home from spirits using protection objects and herbs (.83)

*Reverse scored items

### Demographic differences

Owing to the somewhat mixed research suggesting a correlation between paranormal beliefs, academic discipline and aspects of thinking, responses to the paranormal scale were compared for those with and without higher education backgrounds; and between those from science and non-science academic disciplines. Most participants held an undergraduate degree or higher (n = 164), while less than half held post-secondary qualifications or lower (n = 67). Participants with university degrees had lower total paranormal scores (*M* = 46.20, *SD* = 22.89) than participants without university degrees (*M* = 61.34, *SD* = 22.08). The difference in scores between the two education groups was significant [*t*(126.78) = −4.68, *p* < 0.001]. Of the participants with degree qualifications, most were from science-based disciplines including psychology, natural sciences, technology, and other medical backgrounds (n = 83), while the rest included social sciences, education, business, philosophy, theology, art and humanities, law, and architecture (n = 57). As 24 participants did not disclose their discipline, the following analyses were conducted on 140 participants. Those from science-based disciplines demonstrated lower paranormal scores (*M* = 40.02, *SD* = 21.28) compared to those with art-based degrees (*M* = 54.77, *SD* = 22.24), and the difference in scores between the two discipline groups was significant [*t*(116.99) = 3.92, *p* < 0.001].

### Test–retest reliability

#### Sample and procedure

A follow-up study was conducted to assess the test–retest reliability of the newly developed scale. Of the original sample of 231 participants, 37 (16% of the original sample) agreed to complete the scale a second time, one-week after their initial participation. The retest sample consisted of 21 males (56.80%) and 16 females (43.20%), aged between 18 and 73 (*M* = 41.51, *SD* = 16.61). In contrast to the original sample, this sample had a higher percentage of male participants and a higher mean age. The difference in gender between the original participant group and the retest group was significant (χ^2^ = 5.433, *p* = 0.020). However, the difference in age between the two groups was not significant [*t*(262) = −1.77, *p* = 0.078].

Nineteen respondents were identified as ‘sceptics’ (51.35%) and 18 as ‘believers’ (48.65%), according to their mean scores on the 14-item scale at time one (with those scoring below the overall mean of 50.59 identified as ‘sceptics’ and those above as ‘believers’). The questionnaire completed by participants comprised the original collection of 29 statements and used the same 7-point Likert response format (Strongly Disagree, Moderately Disagree, Slightly Disagree, Uncertain, Slightly Agree, Moderately Agree, Strongly Agree). Responses were numerically coded as before. The scale was administered again as an online survey using Qualtrics Survey Software (Qualtrics, Provo, UT; see https://www.qualtrics.com).

#### Retest analysis

**Fig. 3:**
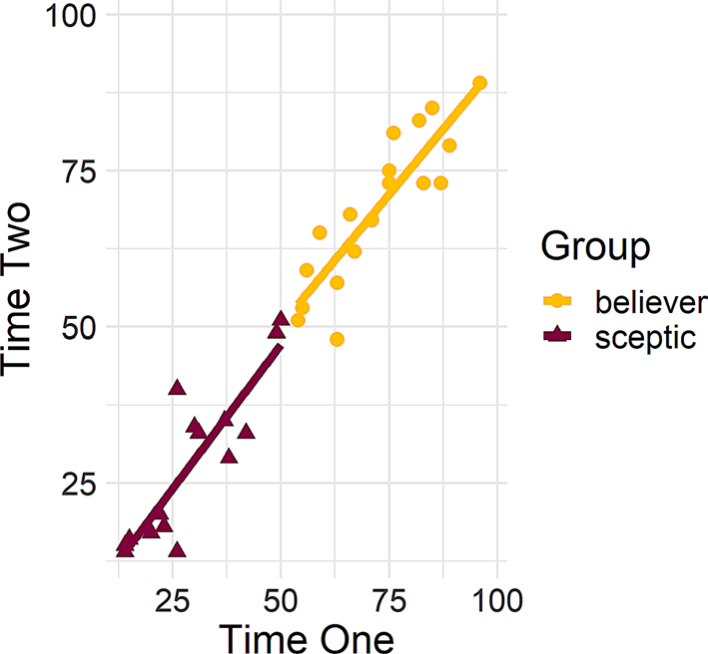
Test–retest reliability analysis as a function of respondent group. Pearson’s correlations between participants’ individual total scores at time one and time two shown for each group

Retest analyses were conducted on the final 14-item scale. Pearson’s correlations revealed a strong test–retest reliability for the scale [*r*(35) = 0.98, *p* < 0.001], as well as for both believers [*r*(15) = 0.88, *p* < 0.001] and sceptics [*r*(18) = 0.90, *p* < 0.001]. A scatterplot of the scores for believers and sceptics at time one and time two can be found in Fig. 3.

## Results: modern test theory

The MTT analyses presented in the following sections were conducted using a Rasch rating scale model (RSM) using the eRm [104, 105] package in R version 4.0.2 [103].

### Response categories

MTT analyses first focused on evaluating the effectiveness of the 7-point Likert rating scale. As it is difficult to be certain of the exact way the sample will use the rating scale, investigation is necessary to verify or improve the functioning of the rating scale categories [106]. To evaluate the response category use of the sample, threshold parameters of each category were examined for each of the original 29 items. These thresholds identify and define the boundaries between each response category and should therefore increase monotonically. Consequently, participants with higher levels of paranormal beliefs should be more likely to endorse higher response categories. For the Rasch analyses, responses are shifted such that the lowest category (strongly disagree) is 0.

Analysis of the 7-point rating scale revealed that threshold parameters failed to increase monotonically, therefore indicating evidence of step disordering. Step disordering, occurring when threshold parameters fail to increase monotonically, indicates that certain response categories have a low probability of being observed [106], meaning that the sample are less likely to use these response categories. The lack of ordered increase occurred at Category 2 (somewhat disagree). Examination of the item category curves (ICCs) indicated that Category 2 had the lowest probability of observance and was therefore never more likely to be observed than any other category. Put more simply, regardless of an individual’s level of belief in paranormal phenomena, the probability of choosing “somewhat disagree” is never the most likely. Similarly, Category 1 (moderately disagree) also had a low probability of observance and at no point was this category most likely to be observed.

**Fig. 4:**
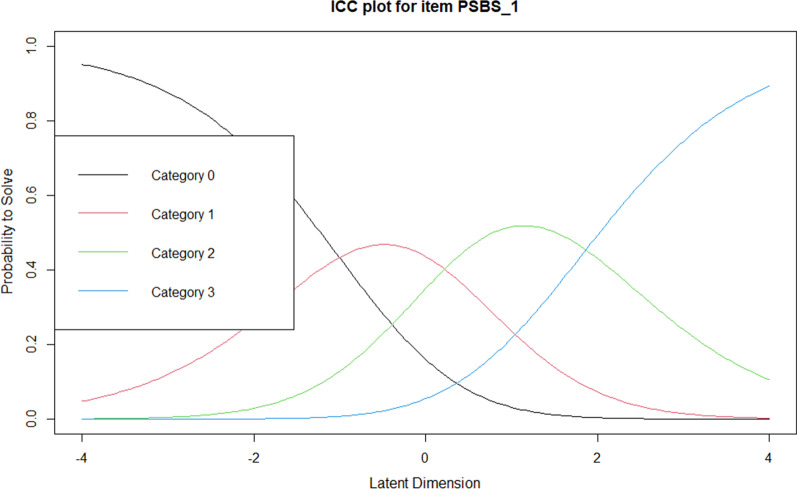
Item characteristic curve for item 1 using the 4-point scoring method. Curves represent the probability of selecting a category along the latent trait. Category 0 = “strongly disagree”, Category 1 = “disagree”, Category 2 = “agree”, Category 3 = “strongly agree”

To begin to improve the functioning of response categories, responses were recoded such that the “moderately disagree” and “somewhat disagree” categories were collapsed, as were the “moderately agree” and “somewhat agree” categories. This gave a revised 5-point scoring method (0 = strongly disagree, 1 = disagree, 2 = uncertain, 3 = agree, 4 = strongly agree). However, this revised scoring method failed to rectify step disordering. Examination of the ICCs revealed that the boundaries between Categories 1 and 2 (disagree and uncertain) were very narrow and suggested that the sample did not clearly differentiate between these two categories. Therefore, a final recoding took place such that the “disagree” and “uncertain” categories were collapsed, giving a final revised 4-point scoring method (0 = strongly disagree, 1 = disagree, 2 = agree, 3 = strongly agree). When this final scoring method was used, the four categories increased monotonically, with the desired appearance of the range of peaks for each category appearing in the ICCs for each item. An example of the ICC for item 1 is shown in Fig. 4.

#### Item fit

Mean square statistics (MNSQ) were computed to determine item fit to the model (i.e., how well each item contributes to defining a single unidimensional construct). The MNSQ statistics indicate the amount of distortion of the scale, where high MNSQ values indicate unpredictability and a lack of construct similarity with other scale items (underfitting), and low values indicate item redundancy and less variation in the observed data compared to the variation that was modelled (overfitting) [107]. Two MNSQ statistics were used to assess item fit: infit (weighted) and outfit (unweighted) statistics. Subsequent analyses used an accepted range of fit of 0.7 to 1.2 [101] to identify items with poor model fit. Therefore, items with MNSQ values < 0.7 were identified as overfitting the model, and MNSQ values > 1.2 were identified as underfitting the model. When assessing item fit to the model, infit and outfit t-statistics were also examined where t-values < -2 were identified as overfitting and t-values > 2 were identified as underfitting. However, it has been suggested that infit and outfit MNSQ values are relatively insensitive to sample size variation in polytomous data, while the t-statistics vary considerably with sample size. Therefore, it has been recommended that infit and outfit t-statistics are interpreted with caution when determining item fit to the model for large samples and polytomous data [101]. As such, items would be removed from the scale if they demonstrated both infit and outfit MNSQ values that were overfitting or underfitting the model. In cases where items were only identified on one of the MNSQ values (infit *or* outfit), t-statistics were consulted to verify item misfit. Based on the MNSQ values of the 29 items, a total of 7 items (4, 10, 12, 13, 15, 28 and 29) were identified for overfitting and a further 8 items (1, 2, 5, 8, 14, 17, 23 and 27) were identified for underfitting. Subsequently, these 15 items were removed from the scale and the analysis was conducted again on the remaining 14 items. A final item (7) was identified for overfitting the model and was removed from the scale. Analysis of the final 13 items revealed infit and outfit statistics within the specified ranges. While item 11 produced an infit t-statistic of − 2.2, the infit and outfit MNSQ values were within the specified range (0.81 and 0.83, respectively) as was the outfit t-statistic (− 1.76). Considering these other statistics and given that the infit t-statistic of item 11 was very close to -2, it was determined that the item demonstrated reasonable fit to the model and that there was not sufficient evidence to remove the item from the final scale. Table 5 shows the final MNSQ statistics for the remaining items, along with the corresponding item difficulty statistics.

**Table 5:** Parameter values for the remaining 13 items (in order of item difficulty)

Item	Outfit MSQ	Infit MSQ	Difficulty
6 If you break a mirror, you will have bad luck	1.002	1.112	2.547
18 Fairies and similar beings are real	1.001	0.866	2.432
19* Fortune tellers’ predictions are typically based on guesswork	1.046	0.823	2.026
16 A person’s star sign can have a direct influence on their personality	0.971	0.993	1.663
21 Some health conditions can be treated with psychic healing	0.986	0.937	1.587
26 It is possible to become possessed by an evil supernatural entity	1.018	0.991	1.540
25* Communicating with spirits or other supernatural entities through a Ouija board is not possible	1.199	1.170	1.475
11 Mind reading is possible	0.832	0.809	1.401
9 It is possible to be reincarnated	1.049	0.974	1.318
22 In some cultures, shamans or “witch doctors” exercise powers we cannot explain	0.892	0.862	1.199
20* Reports of an apparent sixth sense are generally based on fantasies	0.829	0.833	0.829
3 Your mind or soul can leave your body	1.074	1.058	0.793
24 Having a dream that comes true is not just a coincidence	0.832	0.837	0.766

**Fig. 5:**
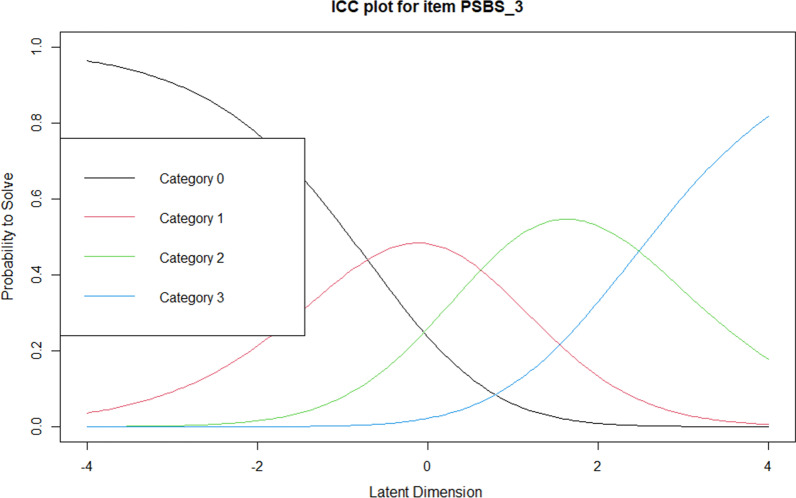
Item characteristic curve for item 3 in the reduced scale using the 4-point scoring method. Curves represent the probability of selecting a category along the latent trait. Category 0 = “strongly disagree”, Category 1 = “disagree”, Category 2 = “agree”, Category 3 = “strongly agree”

Owing to the substantial change in the number of scale items, thresholds for the 4-point response scale were consulted to verify the functioning of the new rating scale for the remaining 13 items. The analysis demonstrated that the thresholds of the four categories increased monotonically for all remaining items. An example of the ICC for item 3 is shown in Fig. 5, which again shows the desired range of peaks.

#### Item difficulty

**Fig. 6:**
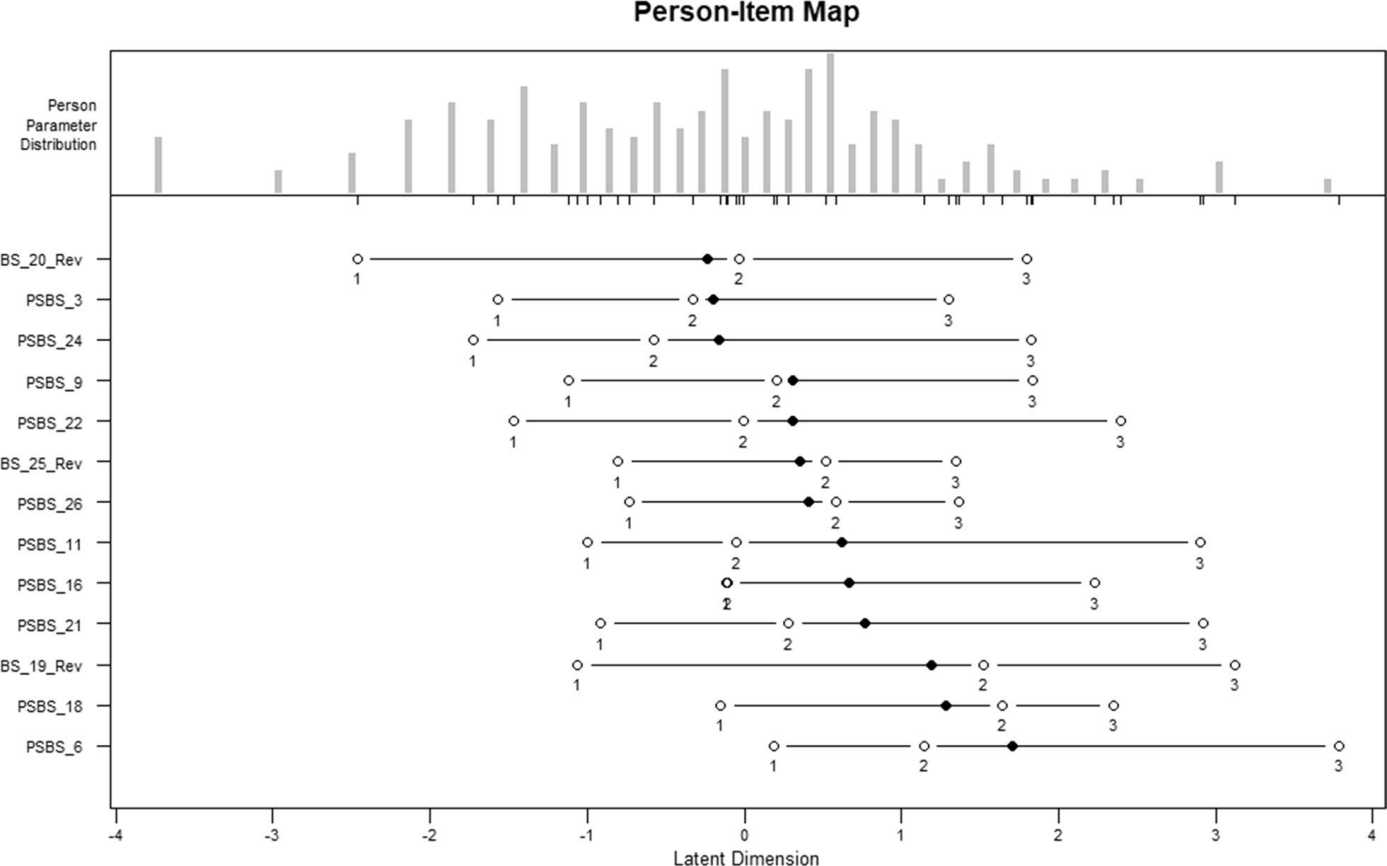
Person-item map for the 13-item scale. Figure displays the location of person traits and item difficulties along the same latent dimension (paranormal belief). The person traits are located on the scale from left (low belief) to right (high belief). Locations of item difficulties are presented as solid circles, and thresholds of adjacent category locations are presented as open circles. The item parameters are located on the scale from least difficult (left) to most difficult (right)

The final RSM analysis conducted using the and eRm package [104, 105] sought to estimate the person trait and item difficulty parameters. In other words, the following analysis aimed to determine whether the difficulty of the remaining items was appropriate for the sample. To meaningfully measure the ability (level of paranormal belief) of all persons, items should be located along the length of the latent dimension. The person-item map shown in Fig. 6 displays both the person traits (in the upper panel) and item difficulties (lower panel) along the same latent dimension. As shown, the category thresholds of most of the 13 items cover a low-to-high range of paranormal belief well. However, item difficulty locations (identified in Fig. 6 as solid circles) cluster towards the right side of the latent dimension. Therefore, the items have a higher probability of differentiating between individuals with higher levels of paranormal beliefs. For example, item 6 (“if you break a mirror, you will have bad luck”) shows the highest item difficulty meaning that participants with higher levels of paranormal beliefs are more likely to agree with this item.

#### Differential item functioning

**Fig. 7:**
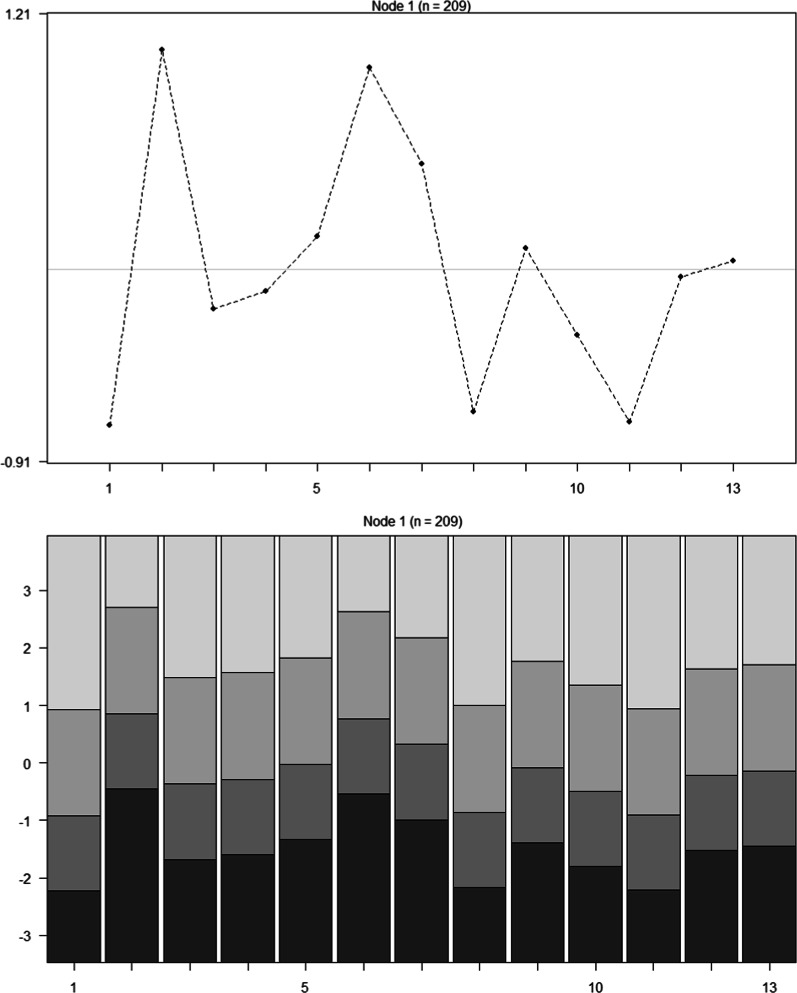
Single-node Rasch tree. *Note: Figure shows differential item functioning analysis conducted on the covariates of age, gender (male or female), ethnicity (white background or BME background), education, and discipline. No differential item functioning was identified. Item number is represented on the x axis in both plots. Item difficulty is represented on the y axis of the top plot (higher values represent higher item difficulty), and item threshold parameters are shown on the y axis of the lower plot (with the lightest shade representing the ‘strongly agree’ response category)*

Differential item functioning (DIF) analysis was conducted using rating scale trees within the psychotree [108, 109] package in R version 4.0.2 [103]. Before this analysis was conducted, data for 8 participants who chose not to disclose demographic information were removed. Data was also removed for participants scoring only in either the highest or lowest categories (i.e., participants responding “strongly disagree” to all 13 items, or “strongly agree” to all items”) as these responses do not provide information relating to item difficulty and therefore do not contribute to the Rasch model. Consequently, data for 14 participants (all of whom scored in the lowest categories) were removed. In total, 22 participants were removed and the DIF analysis was conducted on a reduced sample of 209 participants. If none of the scale items show evidence of DIF, then the analysis should produce a tree with only a single node, supporting a unidimensional Rasch model for the data [110]. However, if the Rasch tree shows at least one split and identifies more than a single node containing the entire sample, then DIF is present. An advantage of using the Rasch tree method for identifying DIF is that DIF can be detected between groups of participants created by more than one covariate (e.g., females under 34), and these groups do not need to be pre-specified prior to analysis. As such, the Rasch tree method searches for the value corresponding to the strongest parameter change and splits the sample at the value identified [110]. The DIF analysis was conducted for five covariates: age, gender, ethnicity, education, and discipline. Analysis produced a tree with a single node, and therefore no DIF was present in the scale for any of the covariates. The single-node tree can be seen in Fig. 7.

#### Confirmatory factor analysis

As a final test of the unidimensionality of the scale, a confirmatory factor analysis (CFA) was conducted using the lavaan [111] package in R version 4.0.2 [103]. To determine the strength of model fit, four main fit indices were used: comparative fit index (CFI), Tucker-Lewis index (TLI), root mean square error of approximation (RMSEA), and standardised root mean square residual (SRMR). For both the CFI and TLI, a value of 0.90 or above would indicate acceptable fit and a value of 0.95 or above would indicate very good model fit. For the RMSEA, a value of 0.05 or below would indicate close model fit, with a value of 0.08 indicating acceptable fit. The accompanying *p* value for the RMSEA statistic should also be greater than the standardised value of 0.05 for close model fit. Finally, an SRMR value of 0.05 or below would indicate a well-fitting model. Overall, the model demonstrated good fit, and supported the use of a unidimensional Rasch model for the data. Complete fit statistics can be seen in Table 5.

### Rasch test–retest reliability

**Fig. 8:**
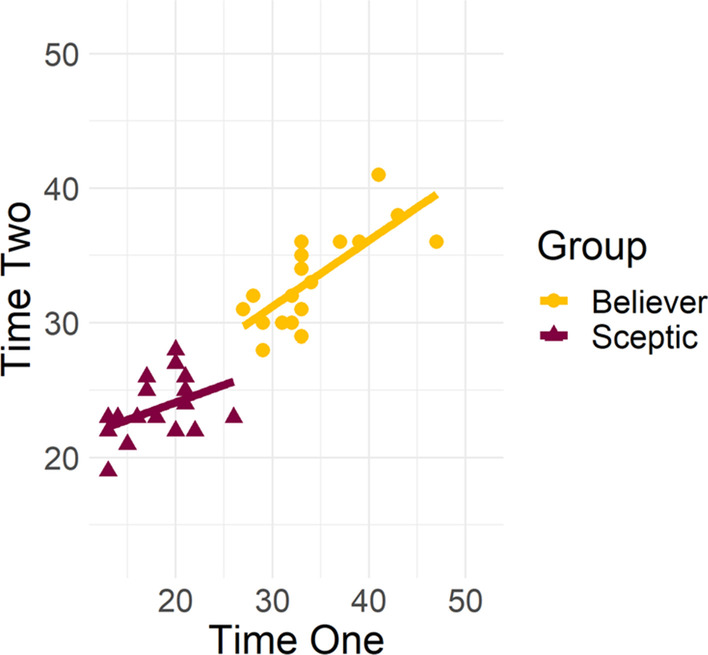
Test–retest reliability analysis for the Rasch scale as a function of respondent group. Pearson’s correlations between participants’ individual total scores at time one and time two shown for each group

The sample for the test–retest reliability analysis was the same as that described in the EFA analysis. While participants were divided into believers and sceptics based on their mean scores for the 13-item Rasch scale at time one (with those scoring below the overall mean of 26.94 identified as ‘sceptics’ and those above as ‘believers’), the analysis retained the original split seen in the EFA analysis of 19 sceptics and 18 believers. Pearson’s correlations revealed a strong test–retest reliability for the scale [*r*(35) = 0.92, *p* < 0.001], and for believers [*r*(16) = 0.75, *p* < 0.001]. However, the retest correlation was not significant for sceptics [*r*(17) = 0.45, *p* = 0.051]. A scatterplot of the scores for believers and sceptics at time one and time two can be found in Fig. 8. Cronbach’s Alpha computed for this final scale, indicated an excellent internal reliability (α = 0.91).

## Correlations between scales

**Fig. 9:**
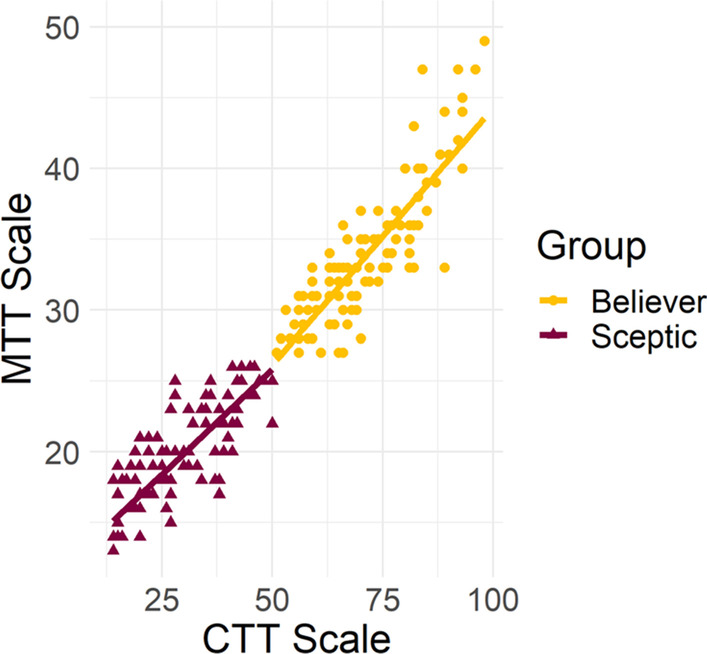
Correlations between respondents’ individual total scores. Pearson’s correlations between respondents’ total scores on the classical test theory and modern test theory scales, as a function of respondent group

To compare the performance of the CTT and MTT derived scales, a final correlational analysis was conducted comparing respondents’ total scores on each scale. The analysis only included respondents who were identified as ‘sceptics’ or ‘believers’ by both scales. Therefore, 17 respondents were removed from the analysis owing to the scales placing them in different groups, and the final analysis was conducted on a reduced sample of 214. Of the reduced sample, 102 respondents were identified as ‘sceptics’ (47.66%) and 112 as ‘believers’ (52.34%). Pearson’s correlations revealed a strong correlation between the scales for the total sample [*r*(212) = 0.96, *p* < 0.001], as well as for both believers [*r*(110) = 0.86, *p* < 0.001] and sceptics [*r*(100) = 0.82, *p* < 0.001]. A scatterplot of the scores for believers and sceptics at time one and time two can be found in Fig. 9.

## Discussion

With a view to developing a new measure of belief in paranormal phenomena, two methods of scale development were compared. The first approach was based on the procedures of classical test theory (CTT), with a particular emphasis on exploratory factor analysis. The second approach used modern test theory (MTT) based on Rasch analysis for polytomous data. The CTT method reduced the initial collection of 29 items to a 14-item scale, describing paranormal belief on a single dimension: *Supernatural Beliefs*. MTT analyses produced a final collection of 13 items measured with a reduced 4-point scoring method. The final MTT derived scale is put forward as the new self-report measure of paranormal and supernatural beliefs, referred to as the ‘Paranormal and Supernatural Beliefs Scale’ (PSBS; see Additional file 1: Paranormal and Supernatural Beliefs Scale).

Several similarities can be seen between the CTT and MTT derived scales. First, both scales support a unidimensional measure of belief in paranormal phenomena. In the CTT analyses, Factor 2 (*Bad Luck*) initially demonstrated an excellent internal reliability. However, examination of the group answering patterns presented interesting findings, with over half of the believers’ responses to these items falling under the “disagree” category. The high “disagree” scores seen for believers in Factor 2 suggest that bad luck may not be diagnostic of belief in more general paranormal phenomena, as the factor was less effective in separating believers and sceptics. For this reason, the three items contained within Factor 2 were removed from the CTT scale. The three items contained within Factor 3 (*Psi*) were also removed from the CTT scale as the factor did not meet satisfactory thresholds (which may be attributed to the fact that all items within this factor were negatively phrased) [112–114]. When initial analyses indicated three distinct categories of belief, the *Supernatural Beliefs* factor explained the most variance and included 70% of the total scale items. This factor was retained as the only factor for the 14-item CTT scale (α = 0.95), and encompassed many phenomena considered to be paranormal or supernatural [115, 116] suggesting that belief in the paranormal may be best characterised by a single overarching factor that is equally understood by both paranormal believers and sceptics. This provides further support for the removal of Factors 2 and 3 from the CTT scale which, while both having their own strengths and weaknesses, may represent categories of beliefs that are separable from paranormal beliefs. Item infit and outfit mean square (MNSQ) statistics (as well as differential item functioning analysis) produced through MTT analyses also indicated that the data supported a unidimensional structure, providing further support for the idea that belief in the paranormal may be best represented by a single dimension. As previous work has suggested a combination of CTT and MTT techniques for psychometric assessment [97], confirmatory factor analysis and reliability analysis (Cronbach’s alpha) were also computed to assess the functioning of the MTT scale items as a complete unit. These findings again supported the unidimensional structure of the scale and indicated an excellent internal reliability (α = 0.91). In addition to high internal reliabilities, both scales demonstrated strong test–retest reliability correlations (0.98 for the CTT scale and 0.92 for the MTT scale). However, examination of the retest statistics for each group (believers and sceptics) revealed differences between the two scales. While the CTT and MTT scales both demonstrated good retest correlations for believers (0.88 and 0.75 respectively, *p*s < 0.001), the retest correlation for sceptics was not significant in the MTT scale [*r*(17) = 0.45, *p* = 0.051] compared to the CTT scale [*r*(17) = 0.90, *p* < 0.001]. The difference in these scores can be explained using the person-item map produced during MTT analyses, which suggested that the item within the MTT scale have a lower probability of differentiating between individuals with lower levels of paranormal beliefs. Similar differences were not able to be established through CTT analyses. To the authors’ knowledge this is the first presentation of separate retest scores for believers and sceptics. Comparison of the performance of both scales revealed strong correlations between respondents’ total scores on the CTT and MTT derived scales in the total sample (*r* = 0.96), and for believers (*r* = 0.86) and sceptics (*r* = 0.82) separately. A final similarity between the two scales can be seen in their item content, as both scales shared 7 common items (approximately half of the total scale content).

Despite the strengths of the CTT scale, and its similarities to the MTT scale, the results of the study provide strong evidence to support preference of the MTT derived scale. First, MTT analyses allowed for investigation and refinement of the 7-point Likert scale. The results indicated that respondents did not require so many response options, and supported removal of three categories leading to a final 4-point scale (1 = strongly disagree, 2 = disagree, 3 = agree, 4 = strongly agree). Categories 1 and 2 of the original Likert scale (moderately disagree and somewhat disagree), both had low probabilities of observance and were subsequently collapsed into a single category (as were the moderately agree and somewhat agree categories). The “uncertain” category was also found to be inadequate in representing participants’ responses, with results suggesting that this category may be poorly defined with respondents not clearly differentiating between this category and the “disagree” category. A 7-point Likert scale was initially selected for the scale as it was thought that the large number of response options would produce a more precise index of respondents’ level of agreement. However, these findings suggest that the response options provided in the original 7-point scale did not represent differentiable levels of belief intensity (as is indicated by a monotonic increase of category thresholds). Additionally, MTT analyses permitted an assessment of differential item functioning (DIF). Using the Rasch tree method for identifying DIF within the MTT scale, analysis focused on five covariates (age, gender, ethnicity, education, and discipline) to determine whether these, or some combination of these, influenced participants’ responses to the scale. Examination of the tree revealed a single node, with no DIF identified for any of the covariates. Therefore, while the MTT scale can be described as a valid measure of belief in paranormal phenomena, it is difficult to be certain that the CTT derived scale does not suffer from DIF. As mentioned above, MTT analyses also allowed for examination of item difficulty, with results indicating that items had a higher probability of differentiating between respondents with moderate-high levels of paranormal beliefs. This information is particularly useful for future research looking to utilise the scale to examine group differences within paranormal beliefs. The following comparisons focus on the final PSBS developed through MTT analyses.

Several important differences can be noted when comparing the PSBS to the three most frequently employed measures of paranormal belief. The unidimensional structure of the PSBS is far simpler than the 7-factor RPBS, with the content of many RPBS factors (such as those within *Witchcraft*, *Spiritualism* and *Precognition*) appearing in the PSBS. The appropriateness of this solution accords with previous research suggesting that a larger array of factors may not provide the most prudent account of paranormal belief [117], particularly as the RPBS has an insufficient number of items to adequately sample seven distinct dimensions of paranormal belief. Such criticisms may explain why a range of studies have failed to replicate the original factor structure of the RPBS, finding smaller factor structures ranging between one and six to be more suitable [117]. Despite this, most of these replication studies have suggested paranormal belief to be a multidimensional construct, which contradicts the findings from the present work. While the structure of the PSBS is more comparable to that of the ASGS (but still differs in terms of dimensionality of belief), the range of items contained within the PSBS is much broader as its focus is not confined to parapsychological phenomena such as extrasensory perception and psychokinesis, though it does include several psi-related items.

The item content of the PSBS also differs considerably from the existing scales in that the final scale presents three negatively phrased items, and contains few cryptozoological, religious, or culturally-specific items. By reducing the number of potentially problematic items and ensuring a blend of positive and negative items, the PSBS reduces the risk of biases introduced by participant response patterns and cultural differences, which have been highlighted as issues for older measures. While cultural differences are often present in paranormal beliefs [118], and consequently some PSBS items have seen cultural influence, the PSBS has a reduced number of culture-bound items compared to previous scales such as the RPBS. Therefore, the PSBS may be a stronger candidate for a universal measure of paranormal belief. A further strength of the PSBS seen particularly when compared to the RPBS, is that that the scale is not affected by certain subgroup characteristics, including respondents’ age gender, ethnicity, level of education, or academic discipline. DIF analysis indicated that the PSBS is a reliable unidimensional scale that can be used to explain data from all respondents. The results seen for the DIF analysis are worth comparing to the RPBS, which contains items that are particularly sensitive to age and gender differences [56], as they suggest that the items within the PSBS have a universal application for respondents regardless of the highlighted subgroups.

Finally, there are a few limitations of the present study which should be noted. First, many of the participants involved in the study were young, well-educated, white females. While analyses confirmed that age, gender, ethnic and educational differences (including academic discipline) do not influence item functioning, further research could explore the psychometric properties of the PSBS with more varied samples and across a diverse range of cultures. Furthermore, although the PSBS focuses on many phenomena that might have a universal application in practice (e.g., communication with spirits), it does present some specific examples that may be more prominent in Western cultures (e.g., Ouija boards). Finally, MTT analyses expressed that the scale is good at measuring moderate-high levels of paranormal beliefs, and so operates sufficiently for the purpose of identifying individuals with increased levels of paranormal beliefs. However, additional items that tap specifically into low levels of paranormal beliefs may be beneficial to add to the scale in future revisions to accurately capture the complete range of beliefs.

### Conclusions

Both CTT and MTT derived scales supported a unidimensional view of belief in paranormal phenomena. However, the scale developed through the MTT model was selected as the final measure owing to the in-depth statistical analyses and refinement this model provided. Although future revisions and further scrutiny of the scale across different samples is warranted, the data and analyses presented here support the psychometric reliability of this new scale for assessing belief in paranormal phenomena. The PSBS displays excellent internal reliability and retest statistics for believers and the total sample, and resolves many of the psychometric and conceptual limitations associated with existing scales. However, it is important to note that the scale is most effective at differentiating between individuals with higher levels of belief. We hope that the PSBS will contribute to future empirical research in the field and provide a universal and reliable alternative to the existing measures of paranormal beliefs currently in use.

## Supplementary Information

**Additional file 1**. Paranormal and Supernatural Beliefs Scale.

## Data Availability

The dataset for the corpus of 29 items generated and analysed during the current study (with the original 7-point response format) is available in the Open Science Framework (OSF) repository, https://osf.io/qvzjy/.

## Abbreviations

RPBS: Revised Paranormal Belief Scale
ELF: extraordinary lifeforms
TRB: traditional religious beliefs
ASGS: Australian Sheep-Goat Scale
SSUB: survey of scientifically unaccepted beliefs
DIF: differential item functioning
CTT: classical test theory
MTT: modern test theory
EFA: exploratory factor analysis
RSM: rating scale model
ICC: item characteristic curve
MNSQ: mean square
CFA: confirmatory factor analysis
PCA: principal components analysis
PSBS: Paranormal and Supernatural Beliefs Scale
