# Supplementary material for: Development of the Paranormal and Supernatural Beliefs Scale using classical and modern test theory
Source: BMC Psychol. 2021 Jun 23;9:98. doi: 10.1186/s40359-021-00600-y (PMC8220838; doi:10.1186/s40359-021-00600-y)
Supplement: Supplementary file 1 — Additional file 1. Paranormal and Supernatural Beliefs Scale. [file 40359_2021_600_MOESM1_ESM.pdf]

Paranormal and Supernatural Belief Scale

The following questions concern your beliefs about paranormal phenomena. There are no right or wrong answers. This is a sample of your own beliefs and attitudes. Please be honest in your responses. Thank you.

0 = Strongly Disagree 1 = Disagree 2 = Agree 3 = Strongly Agree

1. Your mind or soul can leave your body.
2. If you break a mirror, you will have bad luck.
3. It is possible to be reincarnated.
4. Mind reading is possible.
5. A person’s star sign can have a direct influence on their personality.
6. Fairies and similar beings are real.

7*. Fortune tellers’ predictions are typically based on guesswork.

8*. Reports of an apparent sixth sense are generally based on fantasies.

9. Some health conditions can be treated with psychic healing.
10. In some cultures, shamans or “witch doctors” exercise powers we cannot explain.
11. Having a dream that comes true is not just a coincidence.

12*. Communicating with spirits or other supernatural entities through a Ouija board is not possible.

13. It is possible to become possessed by an evil supernatural entity.

Note. Items 7, 8, and 12 are reverse scored.
